# Functional Characterization of *BRASSINAZOLE-RESISTANT 1* in *Panax Ginseng* (*PgBZR1*) and Brassinosteroid Response during Storage Root Formation

**DOI:** 10.3390/ijms21249666

**Published:** 2020-12-18

**Authors:** Hyeona Hwang, Hwa-Yong Lee, Hojin Ryu, Hyunwoo Cho

**Affiliations:** 1 Department of Biology, College of Natural Sciences, Chungbuk National University, Cheongju 28644, Korea; gusa4009@gmail.com; 2 Department of Forest Science, College of Agriculture, Life & Environmental Sciences, Chungbuk National University, Cheongju 28644, Korea; leehy@cbnu.ac.kr; 3 Department of Industrial Plant Science & Technology, College of Agriculture, Life & Environmental Sciences, Chungbuk National University, Cheongju 28644, Korea

**Keywords:** brassinosteroids, *Panax ginseng*, storage root, BZR1, signaling cascade, xylem formation

## Abstract

Brassinosteroids (BRs) play crucial roles in the physiology and development of plants. In the model plant *Arabidopsis*, BR signaling is initiated at the level of membrane receptors, BRASSINOSTEROIDS INSENSITIVE 1 (BRI1) and BRI1-ASSOCIATED RECEPTOR KINASE 1 (BAK1) complex, thus activating the transcription factors (TFs) BRASSINAZOLE RESISTANT 1/BRI1-EMS-SUPPRESSOR 1 (BZR1/BES1) to coordinate BR responsive genes. BRASSINOSTEROIDS INSENSITIVE 2 (BIN2), glycogen synthase kinase 3 (GSK3) like-kinase, negatively regulates BZR1/BES1 transcriptional activity through phosphorylation-dependent cytosolic retention and shuttling. However, it is still unknown whether this mechanism is conserved in *Panax ginseng* C. A. Mayer, a member of the *Araliaceae* family, which is a shade-tolerant perennial root crop. Despite its pharmacological and agricultural importance, the role of BR signaling in the development of *P. ginseng* and characterization of BR signaling components are still elusive. In this study, by utilizing the *Arabidopsis* *bri1* mutant, we found that ectopic expression of the gain of function form of PgBZR1 (*Pgbzr1-1D*) restores BR deficiency. In detail, ectopic expression of *Pgbzr1-1D* rescues dwarfism, defects of floral organ development, and hypocotyl elongation of *bri1-5*, implying the functional conservation of PgBZR1 in *P. ginseng*. Interestingly, brassinolide (BL) and BRs biosynthesis inhibitor treatment in two-year-old *P. ginseng* storage root interferes with and promotes, respectively, secondary growth in terms of xylem formation. Altogether, our results provide new insight into the functional conservation and potential diversification of BR signaling and response in *P. ginseng*.

## 1. Introduction

Brassinosteroids (BRs) are major plant-specific steroid hormones which regulate multiple aspects of growth and development [1,2]. Intensive genetic, biochemical, and physiological studies using the model plant *Arabidopsis* have revealed that BRs play a major role in cell elongation and division, root hair development, seed germination, stomatal development, and various parts of abiotic and biotic interactions through membrane-receptor initiated sequential phosphorylation events, which eventually trigger BR-responsive transcriptional changes [3,4,5,6,7,8,9,10,11,12,13]. In detail, the initiation of BR signaling is tightly mediated by a receptor-like kinase, BRASSINOSTEROID INSENSITIVE 1 (BRI1), and coreceptor kinase, BRI1-ASSOCIATED KINASE 1 (BAK1), at the plasma membrane [14,15]. BR perception through these receptor complex triggers the dissociation of a negative regulator, BRI1 KINASE INHIBITOR 1 (BKI1), and confers transphosphorylation of BRI1 and BAK1, leading to activation of BRI1 SUPPRESSOR 1 (BSU1) and consequent inactivation of BRASSINOSTEROID INSENSITIVE 2 (BIN2) kinase, a representative of the plant glycogen synthase kinase 3 (GSK3) [14,15,16,17,18]. The BIN2 regulates the phosphorylation status of plant-specific transcription factors, BRASSINAZOLE-RESISTANT 1 (BZR1) and BR-INSENSTIVE-EMS-SUPPRESSOR 1 (BES1/BZR2), which play critical roles in BR-perception downstream events via specific binding to the cis-element in the promoter region of large number of target genes [19,20,21]. In the absence of BR, BIN2 is activated by auto-phosphorylation and directly phosphorylates BZR1 and BES1, leading to cytosolic accumulation through 14-3-3 binding and degradation by 26S-proteasome [22,23,24]. Among these well-characterized BR signaling components, the atypical basic helix-loop-helix (bHLH) plant-specific transcription factors BES1, BZR1, and BES1/BZR1 homolog 1-4 (BEH1-4) have been identified as key transcriptional regulators of BR signaling in *Arabidopsis* [22,23,24,25]. These members are functionally redundant in the BR response, including a common set of the binding element in DNA (E-box; CANNTG for BR induced gene expression, BRRE; CGTGT/CG for BR repressed gene expression), and also tightly controlled by BIN2 at the level of post-translational modification [25,26]. Both BES1 and BZR1 proteins physically interact with other transcription factors, epigenetic regulators, and transcriptional repressors. Thus, these protein-protein interactions serve as the platform for crosstalk between BR signaling and other signaling pathways in the context of hypocotyl elongation, flowering, and seed germination during plant growth and development [11,27,28]. These processes are largely dependent on phosphorylation status and nucleocytoplasmic distribution, indicating that canonical BR signaling confers important signaling scaffolds in these cross-talk-driven developmental processes [29]. The primary growth occurs during the initial stages of the plant life cycle, and later, secondary growth additionally provides mechanical strength and generates cells that conduct nutrients and water, supporting the enlarged plant body [30]. Specifically, plant secondary growth is initiated with the formation of the vascular cambium, which continuously provides specialized conducting cells bidirectionally, enabling plants to transport water and nutrients [31]. In recent studies, BR was found to play a key function in the regulation of cambium maintenance and vascular cell differentiation. The core signaling pathway, composed of a peptide/receptor (tracheary element differentiation inhibitory factor; TDIF and TDIF RECEPTOR; TDR/PHLOEM INTERCALATED WITH XYLEM; PXY), WUSCHEL-RELATED HOMEOBOX 4 (WOX4), mediates self-proliferation and differentiation in close interaction with the BR signaling pathway [32,33,34,35]. The TDR-TDIF signaling directly suppresses BES1 and BZR1 through the activation of the BIN2 kinase in the cambial region, which inhibits and facilitates xylem formation and cambium proliferation, respectively [35]. Moreover, BES1/BZR1 and BIN2 act on the fine-tuning of auxin signaling output, which is essential for cambium maintenance and xylem formation at multiple level of controls in its transport and signaling cascade [36,37,38,39,40]. However, the role of BR signaling during the formation of the storage root, the hidden half of the secondary growth of the plant, has not yet been explored. 

In the present research, we isolate the BR signaling components (PgBRI1, PgBIN2, and PgBZR1) in *Panax ginseng* (*P. ginseng*), an important medicinal plant that accumulates various numbers/amounts of saponins (ginsenosides) in the storage root. By utilizing the *Arabidopsis* protoplast transient expression system, we found that the phosphorylation status and consequent nucleus-cytoplasmic redistribution of PgBZR1 were tightly controlled by BR perception and activity of PgBIN2, while the responsiveness upon upstream signal was distinct from that of *Arabidopsis*. In addition, the ectopic expression of the hyperactive form of *PgBZR1* (*Pgbzr1-1D*) restored a broad spectrum of BR-deficiency phenotypes caused by the *bri1* mutation, such as the stem/hypocotyl elongation and flower/silique development, suggesting that BR signaling transcription factor PgBZR1 has a conserved function in *Arabidopsis* and a similar BR signaling mechanism of *P. ginseng*. Notably, the exogenous application of BR and inhibition of BR biosynthesis in *P. ginseng* showed that BR plays an inhibitory role in xylem differentiation in storage root formation. In line with these data, we found that the ectopic expression of *Pgbzr1-1D* strongly suppressed secondary growth in terms of xylem formation in *Arabidopsis*, supporting the notion that BR response through PgBZR1 acts as a suppressor of xylem differentiation. Taken together, we have functionally characterized the *PgBZR1*, its regulation mechanism, and BR response during *P. ginseng* storage root formation.

## 2. Results

### 2.1. Phylogenetic Analysis of Brassinosteroid Signaling Components in P. ginseng

**Figure 1:**
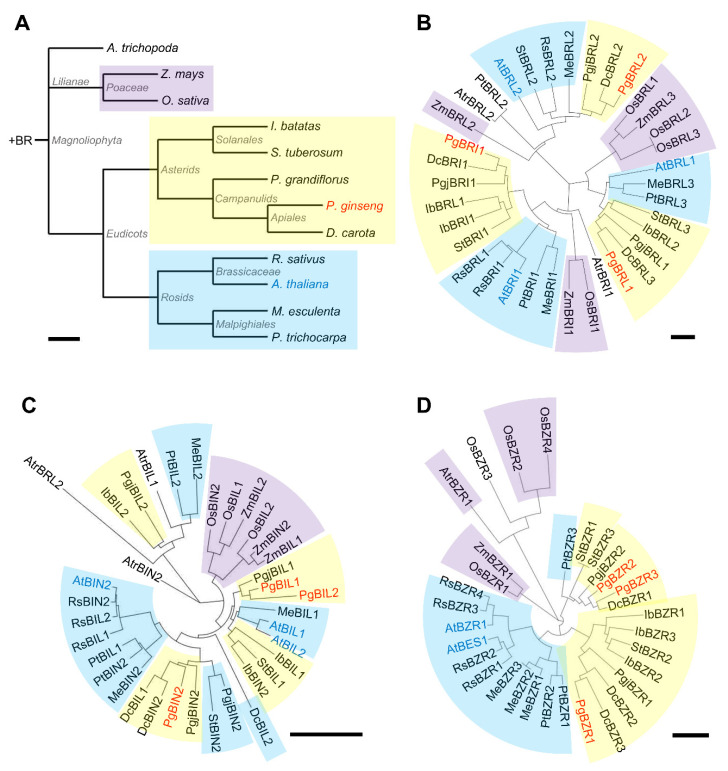
Phylogenetic analysis of BR signaling components in *Magnoliophyta*. (**A**) A schematic diagram of the plant species phylogeny. (**B**–**D**) Phylogenetic trees of BR signaling components in *Magnoliophyta*. The BR signaling components were subtracted from publicly available genome database (https://phytozome.jgi.doe.gov/pz/portal.html) and local blast program using BioEdit (https://bioedit.software.informer.com/7.2/). The ClustalX2 program was used to align the amino acid sequences of each components, and phylogenetic trees were constructed with MEGA 7 program using the neighbor-joining method. Tree scale bars: 0.1. Color code: yellow for *Asteroids*; blue for *Rosids*; purple for *Poaceae*.

A phylogenetic tree analysis was conducted to determine whether the components of the BR signaling mechanism showed a taxonomic relationship within the plant species of *Magnoliophyta* (Figure 1). During plant evolution, plant hormone signaling mechanisms did not appear simultaneously, but emerged at specific branching points. In particular, the signaling mechanisms of gibberellin (GA), jasmonic acid (JA), ethylene (ETH), and BRs evolved after the emergence of vascular plants, and BR signaling mechanisms emerged at the point of divergence of angiosperm (*Magnoliophyta*) and gymnosperm from seed plants [41]. *Magnoliophyta* is subdivided into several subclades, including the most primitive order of *Magnoliophyta*, *Amborella trichopoda* (*A. trichopoda*), monocots, and eudicots (Figure 1A). Both *P. ginseng* and *Arabidopsis* belong to the eudicots but are found in different subclades. *P. ginseng* and *Arabidopsis* are in *Asterids* and *Rosids*, which are the largest subclades of eudicots. This indicates that *P. ginseng* and *Arabidopsis* do not have the closest relationship. Therefore, we selected representative plant species by considering their taxonomic location, phenotypes, and available genome sequence data for further analysis. To dissect evolutionary relationships among BR signaling components, we constructed the phylogenetic trees of major BR signaling components in *Magnoliophyta* (Figure 1B–D). We focused on the major regulators of BR signaling, BRI1, BIN2, and BZR1. All of the components tend to be grouped in line with taxonomic relations. The BRI1 related components are divided into three clades in accordance with protein identity, and further grouped by taxonomic relation (Figure 1B). The BIN2 related components show a distinctive separation between monocots and eudicots, but tend not to have a taxonomic relation among eudicots (Figure 1C). Notably, the BZR components tend to be grouped depending on the taxonomic relationship compared with BRI1 and BIN2 from *Magnoliophyta* (Figure 1D). These results collectively suggest that BZR1 and BRI1 were more diversified, and potentially acquired specialized function in contrast to BIN2 during the evolution of *Magnoliophyta*.

### 2.2. Identification of Arabidopsis BRI1, BIN2, and BZR1 Homologs in P. ginseng

**Figure 2:**
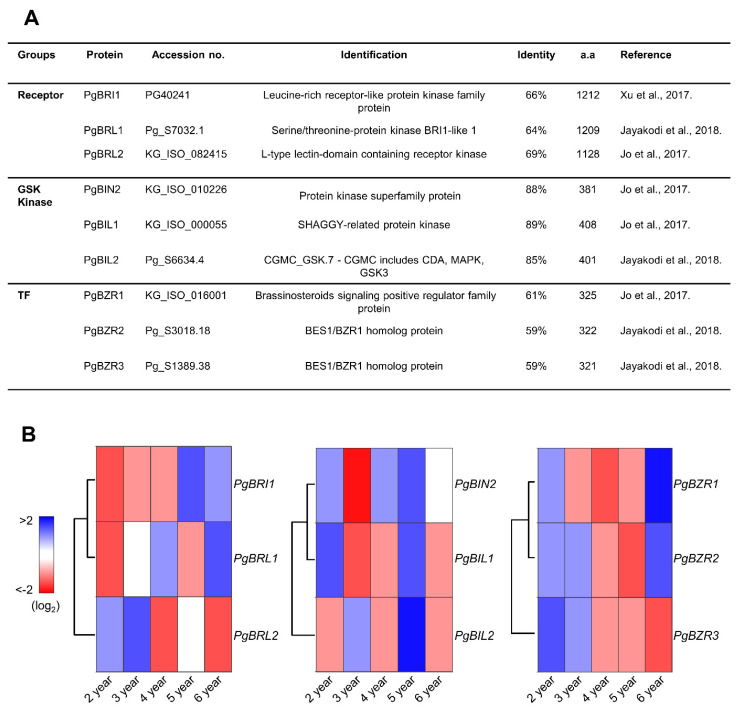
Characteristics of BR signaling components of *P. ginseng* and their expression profiles in *P. ginseng*. (**A**) The accession numbers, sizes, and identities of BR signaling components in *P. ginseng*. (**B**) The heat map of *PgBRIs*, *PgGSKs*, and *PgBZRs* expression levels in different ages of *P. ginseng*. The heat maps were constructed by the XLSTAT program. FPKM (fragments per kilobase of transcript per million reads mapped) values were visualized by red/blue color range with log2 scale.

**Figure 3:**
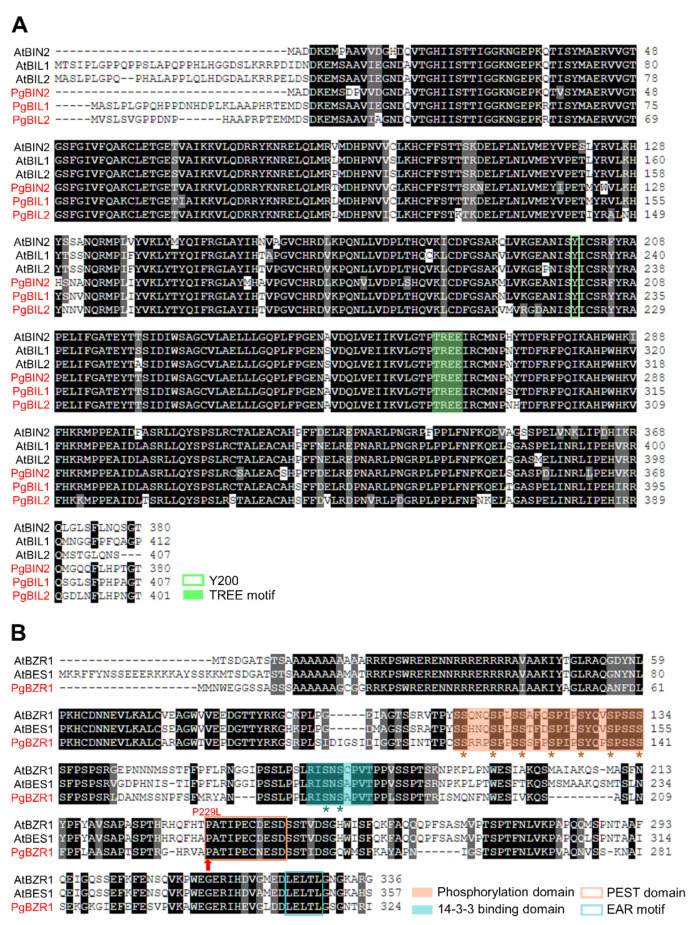
Multiple sequence alignment of PgGSK family and PgBZR1 with *Arabidopsis* proteins. (**A**) The Y200 residue required for BIN2 activation and TREE motif of catalytic domain were completely conserved in PgGSK family. (**B**) The amino acid residues of phosphorylation domain and 14-3-3 protein binding domain (* indicates phosphorylated residues) were conserved in PgBZR1. The red arrow indicates location of point mutation for bzr1-1D gain of function. Identical amino acids were shaded in black, and similar amino acids were shaded in gray. Conserved domains were indicated with different color codes.

We found that the taxonomic position of *P. ginseng* is the closest to *D. carota*. However, due to the lack of functional characterization of BR signaling components in *D. carota*, we searched the homologs of BRI1, BIN2, and BZR1 in *P. ginseng* based on *Arabidopsis* BR studies (Supplementary Figure S1 and Figure 2A). The BR signaling components in *P. ginseng* were extracted using a combination of genome sequence and transcriptome data based on their amino acid sequence identities with *Arabidopsis* BRI1, BIN2, and BZR1 [42,43,44]. We analyzed the phylogenetic tree of potential BRI1, BIN2, and BZR1 in *P. ginseng* in accordance with sequence identity and similarity with *Arabidopsis* (Supplementary Figure S1). We selected representative genes, considering the relationship, protein size, and functional domain structure with *Arabidopsis*. Based on these parameters, we referred to PG40241, Pg_S7032.1, and KG_ISO_082415 as the BR receptor group (PgBRI1, PgBRL1, and PgBRL2), KG_ISO_010226, KG_ISO_000055, and Pg_S6634.4 as the GSK kinase group (PgBIN2, PgBIL1, and PgBIL2), and KG_ISO_06001, Pg_S3018.18, and Pg_S1389.38 as TF group (PgBZR1, PgBZR2, and PgBZR3) (Figure 2A, Figure 3, and Supplementary Figure S2). As shown in the alignment results, PgBRI1 has two central domains: the extracellular domain (ECD) for the perception of BR and the intracellular domain (ICD) for the activation of downstream signaling cascades through phosphorylation. The BR binding site of ECD, the island domain, and the amino acid residues that are important for the kinase activity of ICD are highly conserved in PgBRI1 (Supplementary Figure S2). PgBIN2 has also conserved Y200 amino acid residue and the TREE motif which are important for the activation of BIN2 to phosphorylate the BZR1 (Figure 3A). The highest identity of the GSK kinase group between *P. ginseng* and *Arabidopsis* supports the potential functional conservation of GSK kinases within different plant species, as shown in the taxonomic analysis. Interestingly, despite the low identities (59%–61%) between PgBZR1 and AtBZR1, the former has highly conserved crucial domains and amino acid residues required for BR-dependent activation/suppression, suggesting similar modes of action regulating the BZR1 activity (Figure 3B). 

To assume the BR signaling status during the annual growth of *P. ginseng*, we analyzed the gene expression patterns of identified BR signaling components in different ages of *P. ginseng* (Figure 2B). Interestingly, based on their positive or negative effect on BR signaling, the *PgBRI1* and *PgBIN2* show antagonistic expression patterns in two- and six-year-old *P. ginseng*, which suggests that there is a potential positive correlation between *P. ginseng* age and activation of BR signaling status. Overall, the analysis of conserved domains involved in the regulation of BR signaling suggests the potential of their functional conservation with *Arabidopsis*, and BR component gene expression patterns in *P.ginseng* roots of different ages suggest dynamic BR regulation during *P. ginseng* development.

### 2.3. PgBIN2 Phosphorylates and Leads Nucleocytoplasmic Shuttling of PgBZR1 from the Nucleus to Cytosol

**Figure 4:**
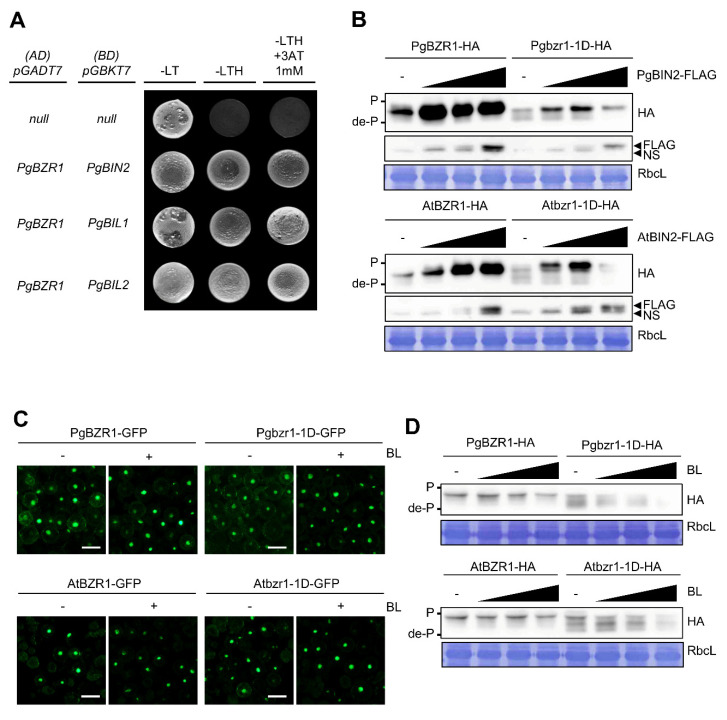
PgBIN2 and BL regulate the phosphorylation status of the PgBZR1. (**A**) PgBZR1 directly interacts with PgGSK family in yeast two-hybrid assay. (**B**) PgBIN2 phosphorylates the PgBZR1 and Pgbzr1-1D. *HA-tagged PgBZR1* or *Pgbzr1-1D* was cotransfected into *Arabidopsis* protoplast with *PgBIN2-FLAG* (upper panel). *HA-tagged AtBZR1* or *Atbzr1-1D* was cotransfected into *Arabidopsis* protoplast with AtBIN2-FLAG (lower panel). After transfection for 6 h, the indicated proteins were visualized with anti-HA or anti-FLAG antibodies. The protein levels were determined by coomassie blue staining (RbcL). Black arrow head indicates the FLAG-BIN2 or non-specific band (NS). (**C**,**D**) BL induces nucleus accumulation and dephosphorylation of PgBZR1 and Pgbzr1-1D, respectively. GFP tagged BZR1 or bzr1-1D from *P. ginseng* (upper panel) or *Arabidopsis* (lower panel) were transfected into *Arabidopsis* protoplast and treated with 10 μM BL for 2 h after 12 h of incubation. Scale bars: 50 μm (**C**). BZR1-HA or bzr1-1D-HA from *P. ginseng* (upper panel) or *Arabidopsis* (lower panel) was transfected into *Arabidopsis* protoplast, and incubated for 6 h with or without 10 μM BL. The proteins were visualized with anti-HA antibody (**D**).

To test whether PgBZR1 is a direct substrate for phosphorylation via PgBIN2, PgBIL1, and PgBIL2, we first performed a Yeast two-hybrid (Y2H) assay to examine the direct physical interactions (Figure 4A). Consistent with direct interaction between group II GSK3s (BIN2, BIL1, and BIL2) and BZR1/2 in *Arabidopsis*, we found that PgBZR1 directly interacted with PgBIN2, PgBIL1, and PgBIL2 in the Y2H system (Figure 4A). We thus examined whether PgBZR1 is directly phosphorylated by PgBIN2 and the degree of phosphorylation status using *Arabidopsis* mesophyll protoplast (Figure 4B). The *hemagglutinin (HA)-fused PgBZR1* or *Pgbzr1-1D* was cotransfected into protoplast with *FLAG-fused PgBIN2* (Figure 4B). PgBIN2 phosphorylated PgBZR1 protein in a dose-dependent manner, but Pgbzr1-1D protein responded less effectively to PgBIN2, and the dephosphorylated form was still accumulated in cells coexpressing PgBIN2 (Figure 4B), indicating that the canonical BIN2 action on BZR1 is also a remnant in *P. ginseng*. By comparison, AtBIN2 coexpressed with AtBZR1 did not fully phosphorylate AtBZR1 and failed to induce the complete phosphorylation of Atbzr1-1D, suggesting higher BIN2 kinase activity of *P. ginseng* than that of *Arabidopsis* (Figure 4B). To examine this hypothesis, we utilized the synthetic BIN2 inhibitor bikinin to quantitatively assess the differential phosphorylation kinetics of PgBIN2 and AtBIN2 (Supplementary Figure S3) [45]. We found that the coexpression of BIN2 induced phosphorylation of BZR1 protein from both *P. ginseng* and *Arabidopsis*, whereas the bikinin treatment more efficiently antagonized the phosphorylation of the *Arabidopsis* BZR1 protein than the PgBZR1 in the presence or absence of BIN2 (Supplementary Figure S3A,B). In addition, the dose-dependent inhibitory effect of bikinin on PgBIN2 kinase activity and its readout PgBZR1 dephosphorylation were less effective compared to the AtBIN2 action on AtBZR1 (Supplementary Figure S3C,D). Next, we examined the effect of BR on the phosphorylation status and subcellular distribution of PgBZR1 and Pgbzr1-1D (Figure 4C,D). The exogenous application of brassinolide (BL) led to the accumulation of the dephosphorylation form and nuclear localization of AtBZR1 and Atbzr1-1D in a dose-dependent manner (Figure 4C,D). However, the phosphorylation form of PgBZR1 protein still remained in the same concentration of BL, which was enough to induce a substantial amount of AtBZR1 dephosphorylation form (Figure 4C), supporting the hypothesis that the differential magnitudes of the BR signaling cascades from the BR perception to the transcription factor of *P. ginseng*. In addition, Pgbzr1-1D protein appeared to be more accumulated in the cytosol in the absence of BL, even with an increased dephosphorylation status compared to the PgBZR1 protein, also suggesting the existence of another layer of phosphorylation-dependent nucleocytoplasmic shuttling mechanism (Figure 4D). 

**Figure 5:**
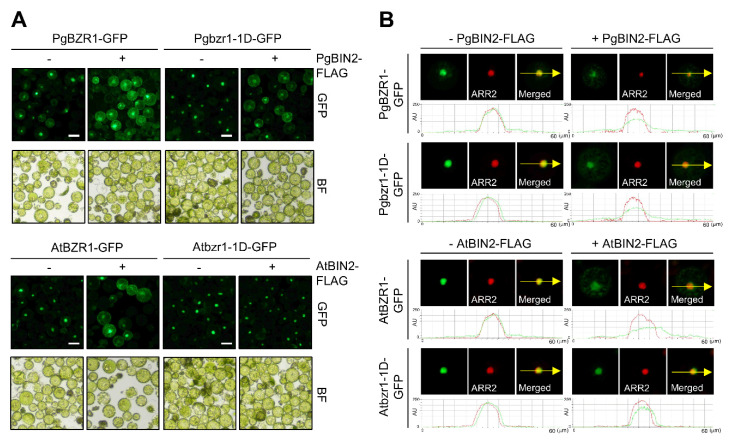
PgBIN2 controls the subcellular localization of PgBZR1 and Pgbzr1-1D by shuttling from the nucleus to the cytoplasm. (**A**) *GFP-tagged PgBZR1* or *Pgbzr1-1D* was transfected into *Arabidopsis* protoplast with or without *PgBIN2-FLAG* for 12 h. BF; bright field. Scale bars: 50 μm. (**B**) *RFP*-*tagged ARR2* was used for nuclear marker and cotransfected with *PgBZR1-GFP*, *Pgbzr1-1D-GFP* with or without *PgBIN2-FLAG*. The intensities of the fluorescent signals from PgBZR1-GFP, Pgbzr1-1D-GFP, or ARR2-RFP were measured using NIS-Elements Basic Research (Eclipse Ts2, Nikon, Japan). The yellow arrows indicate the point of measurement.

To explore the spatial dynamics of PgBZR1 and the role of phosphorylation by PgBIN2 on subcellular redistribution, we focused on the Pgbzr1-1D protein in the presence of PgBIN2 in the protoplast system (Figure 5A,B). As previously reported, AtBZR1 was transported from the nucleus to the cytosol in the presence of AtBIN2. However, Pgbzr1-1D rarely responded by phosphorylation by PgBIN2 at the level of subcellular redistribution from the nucleus to the cytosol (Figure 5A,B). 

### 2.4. Ectopic Expression of PgBZR1 and Pgbzr1-1D Alleviates Stem Elongation in BR-Insensitive Bri1-5 Mutant

**Figure 6:**
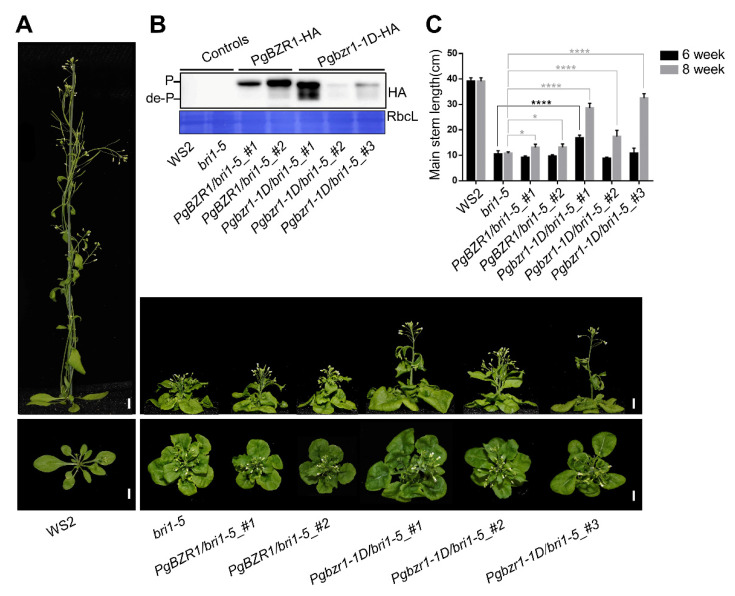
Overexpression of *Pgbzr1-1D* attenuates the dwarfism of *bri1-5*. (**A**) The representative shoot phenotype of five-week-old indicated genotypes. Scale bars: 1 cm. (**B**) The protein levels of PgBZR1 and Pgbzr1-1D in ten-day-old transgenic plants. proteins were visualized with an anti-HA antibody. WS2 and *bri1-5* were used as negative controls. (**C**) The inflorescence stem length of six- and eight-week-old indicated genotypes. (* for *p* < 0.1, **** for *p* < 0.0001 by Two-way ANOVA).

To explore the physiological role underlying canonical BR action on the phosphorylation status of PgBZR1 and consequent BR response, we first generated a transgenic *Arabidopsis* overexpressing *HA-fused PgBZR1* and *Pgbzr1-1D* under the control of C4PPDK 35S promoter in BR-insensitive *bri1-5* mutant background (Figure 6A,B). Among these transgenic lines, *PgBZR1/bri1-5 #2* and *Pgbzr1-1D/bri1-5 #1* showed the strongest accumulation of the phosphorylated and dephosphorylated forms of BZR1, respectively (Figure 6B). All the Pgbzr1-1D overexpression lines showed long inflorescence stem lengths compared with the *bri1-5* mutant, but failed to fully recover the BR-deficient dwarfism (Figure 6A,C). Interestingly, inflorescence stem elongation in wild-type and *bri1-5* mutant was terminated six weeks of age, but the ectopic expression of *Pgbzr1-1D* in *bri1-5* mutant led to a prolonged stem elongation until eight weeks (Figure 6C). Based on the phenotypic analysis of the PgPZR1 and Pgbzr1-1D overexpression effect on BR-defective dwarfism, the partial restoration of stem elongation through PgBZR1 and/or Pgbzr1-1D suggests that PgBZR1 functions as a transcription factor, mediating the BR-induced stem elongation. In addition, *Pgbzr1-1D/bri1-5 #1* and #3 rescue the defect of petiole elongation of bri1-5, implying that PgBZR1 is also involved in petiole development (Supplementary Figure S4). In total, the partial restoration of stem length and petiole elongation of *bri1-5* suggests that PgBZR1 functions as a key transcription factor downstream of BR perception. 

### 2.5. Ectopic Expression of Pgbzr1-1D Restores Inflorescence Architecture and Reproductive Organ Defects of the bri1-5 Mutant

**Figure 7:**
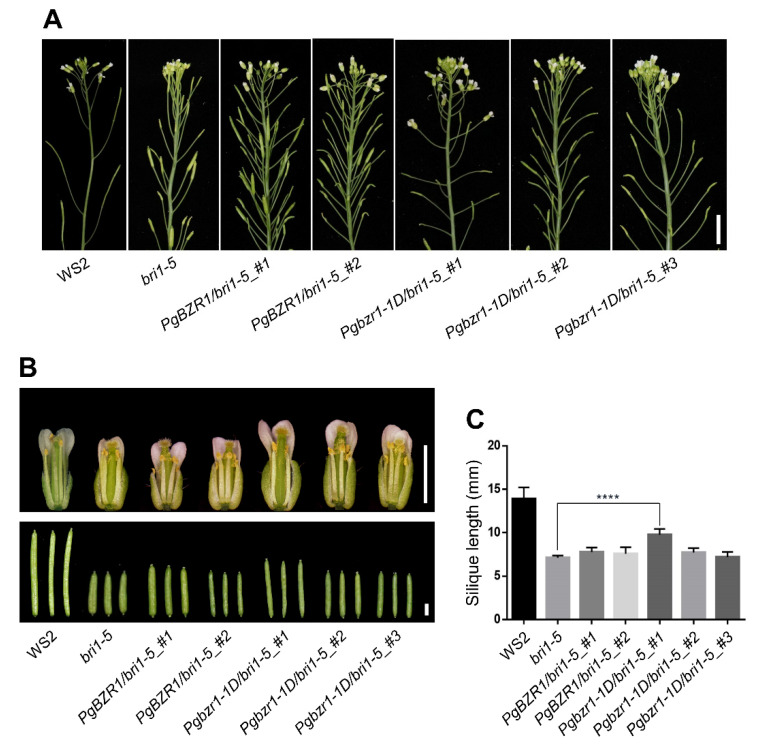
Overexpression of *Pgbzr1-1D* partially rescues the axillary meristem defect, flower development, and silique growth of *bri1-5*. (**A**) The representative axillary meristem formation of six-week-old indicated genotypes. Scale bar, 1 cm. (**B**) The representative flower and silique development of indicated genotypes at eight-week-old. Scale bars: 2.5 mm. (**C**) The quantification of silique length before dehiscence (*n* = 10, **** for *p* < 0.0001, by One-way ANOVA).

In an attempt to further test whether PgBZR1 functions as a BR transcription factor, we investigated the ectopic expression of the *Pgbzr1-1D* effect on reproductive development. In previous studies, BR-deficiency was shown to cause reduced floral organ size [46,47]. In line with the expansion of inflorescence stem and petiole length, the ectopic expression of *Pgbzr1-1D* completely restored the size of the floral organ, especially for petal (Figure 7B). Interestingly, none of the transgenic plants expressing PgBZR1 restored either floral organ size or axillary meristem formation, whereas *Pgbzr1-1D* overexpression partially compromised these defects of bri1-5 (Figure 7A,B) in a protein accumulation dependent manner (Figure 6B). Consistent with these data, the *Pgbzr1-1D/bri1-5 #1* line, which showed the highest accumulation of dephosphorylated PgBZR1 in the *bri1-5* background also restored the short silique length to the wild-type level (Figure 7B,C), indicating that hyper-active PgBZR1-mediated BR signaling is largely required for reproductive development. These results indicate that PgBZR1 acts as a BR signaling transcription factor during floral development and reproductive growth. 

### 2.6. Ectopic Expression of Pgbzr1-1D Compromises the Deficiency of Dark-Induced Hypocotyl Elongation in bri1-5

**Figure 8:**
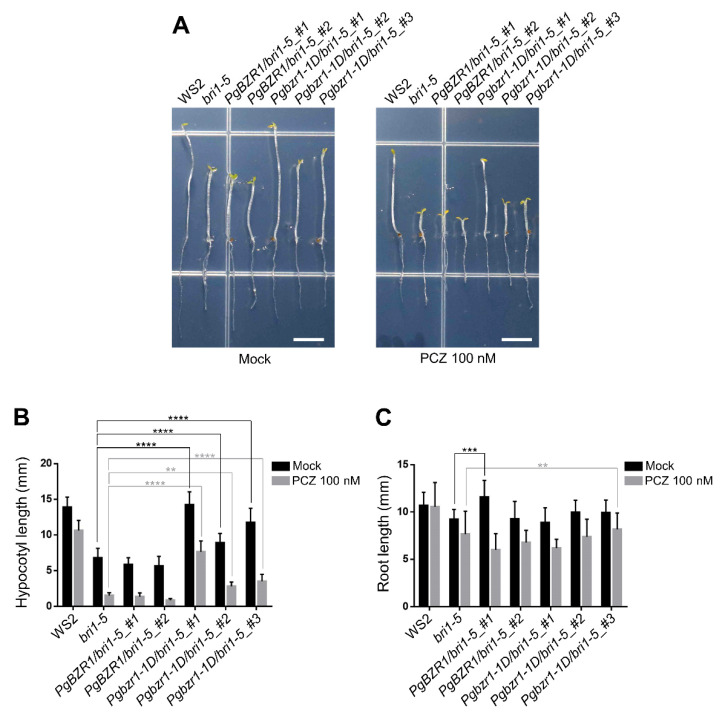
Overexpression of *Pgbzr1-1D* compromises the defect of the dark induced hypocotyl elongation in *bri1-5*. (**A**) The representative hypocotyl elongation phenotype of indicated genotypes grown in the MS media absence or presence of 100 nM PCZ. Scale bars: 5 mm. (**B**,**C**) The quantification of hypocotyl (**B**) or root length (**C**) of 10-day-old seedlings. (*n* ≥ 20 plants, ** for *p* < 0. 01, *** for *p* < 0.001, **** for *p* < 0.0001 by Two-way ANOVA).

Next, we assessed whether PgBZR1 plays a role in hypocotyl elongation, a typical BR response in *Arabidopsis*. To achieve this, we examined dark-induced hypocotyl elongation of *PgBZR1* and *Pgbzr1-1D* transgenic lines in the *bri1-5* background in the absence or presence of propiconazole (PCZ), a specific BR biosynthesis inhibitor. All of the PgBZR1-overexpressing dark-grown seedlings showed similar elongation to the *bri1-5* mutant in both conditions (Figure 8A,B). However, *Pgbzr1-1D* transgenic line #1 conferred a similar elongation of hypocotyl compared to the wild-type in both of conditions (Figure 8A,B). Notably, this line showed significant resistance to the PCZ and longer hypocotyl than *bri1-5* mutant (Figure 8A,B). However, the root growth of all genotypes did not correlate with their BR signaling output and/or PgBZR1 accumulation (Figure 8A,C). The discrepancy of the PgBZR1-mediated output between shoot and root suggests that PgBZR1 action and downstream BR target genes are largely shared with *Arabidopsis* BR response during the primary growth of the aerial parts (leaf, inflorescence stem, flower, silique, and hypocotyl) but might become diversified for root growth and development. 

### 2.7. BR and PgBZR1 Repress Xylem Differentiation during Storage Root Formation of P. ginseng and Secondary Growth of Arabidopsis

**Figure 9:**
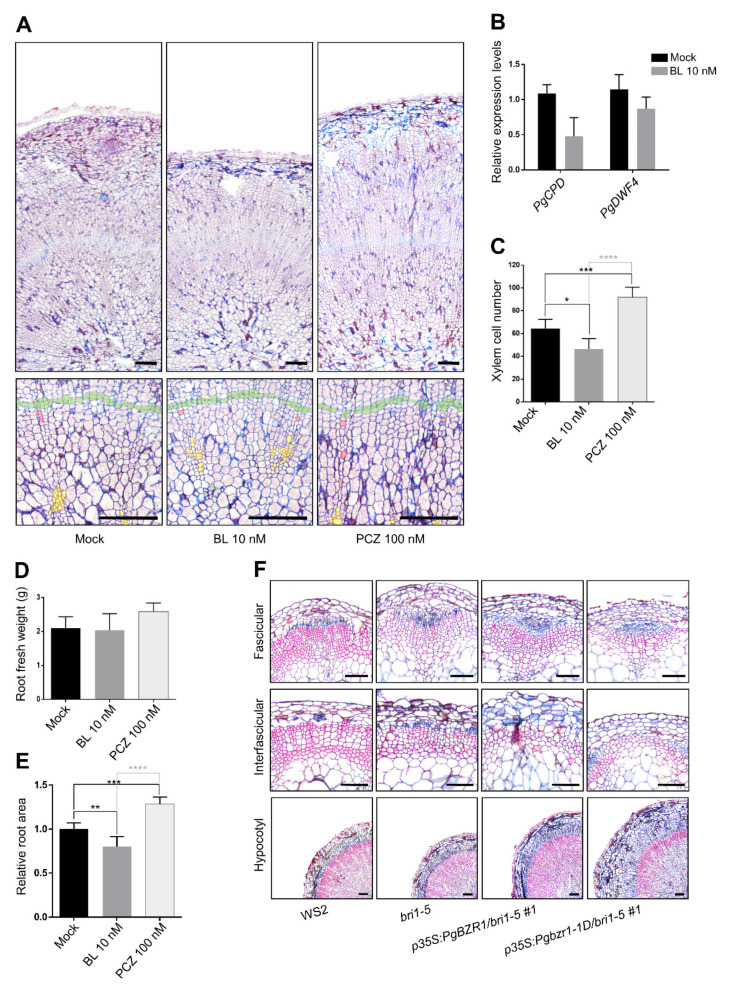
BL negatively regulates the root thickening and xylem formation in *P. ginseng* and overexpression of *PgBZR1* and *Pgbzr1-1D* reduce xylem formation of stem and hypocotyl in *Arabidopsis*. (**A**) The representative cross section images of *P. ginseng* taproots (two years old) treated with 10 nM BL or 100 nM PCZ for 8 weeks. Color code: yellow for xylem regions developed in one-year-old specimens; red for xylem regions newly developed after two years; green for cambium regions. Scale bars: 250 μm. (**B**) The expression levels of *PgCPD* and *PgDWF4* in two-week-old seedlings of *P. ginseng* after treatment of 10 nM BL for 3 h determined by quantitative RT-PCR (*n* = 3, 2 plants per *n* = 1). (**C**) Total number of xylem cells in two-year-old *P. ginseng* that were developed during over two years (*n* = 5 plants, * for *p* < 0.1, *** for *p* < 0.001, **** for *p* < 0. 0001 by One-way ANOVA). (**D**) The fresh weight of *P. ginseng* root after treatment with 10 nM BL or 100 nM PCZ for 8 weeks (*n* = 5 plants). (**E**) The relative area of *P. ginseng* taproot cross section samples which are treated with 10 nM BL or 100 nM PCZ for eight-week-old (*n* = 5 plants, ** for *p* < 0.01, *** for *p* < 0.001, **** for *p* < 0. 0001 by One-way ANOVA). (**F**) The representative cross section images of inflorescence stem or hypocotyl of eight-week-old indicated genotypes. Scale bars: 100 μm. All images were counterstained with 0.5% safranin and 1% astra-blue.

To investigate the role of BR signaling in storage root formation in *P. ginseng*, we monitored the secondary growth of *P. ginseng* root in the presence of BL or propiconazole (PCZ) (Figure 9A). At first, we examined the expression level of *PgCPD* and *PgDWF4* (Supplementary Figure S5), BR-responsive genes in *P. ginseng* seedling after BR treatment to evaluate the physiological concentration of exogenous BR treatment and determine whether consequent BR signal transmission occurred in *P. ginseng* (Figure 9B) [48,49]. An application of 10 nM BL reduced the expression of *PgCPD* as well as *PgDWF4*, indicating that 10 nM of BL has a physiological range of BR treatment for triggering of BR signaling in *P. ginseng*. Then, we performed histological analysis using a two-year-old *P. ginseng* root after the prolonged alteration of BR signaling (Figure 9A,B). BL treatment for eight weeks resulted in a dramatic decrease of newly formed xylem vessel (after reactivation of cambium in the spring season) compared with mock-treated control. By contrast, the inhibition of BR biosynthesis by PCZ treatment stimulated xylem vessel formation during the eight weeks of secondary growth of *P. ginseng*. These pharmacological approaches combined with a histological analysis of vascular tissue strongly suggest that BR signaling negatively regulates xylem vessel differentiation during *P. ginseng* storage root formation (Figure 9A–E). 

We then examined the xylem formation during the secondary growth of *PgBZR1* and *Pgbzr1-1D* overexpressing lines in the *bri1-5* mutant background (Figure 9F). In line with the well-characterized role of BR signaling in xylem differentiation, the *bri1* loss-of-function mutation caused a strong defect in both of inflorescence stem and hypocotyl in the context of the vessel and fiber formation compared with the wild-type (Figure 9F) [35]. Notably, the ectopic expression of *PgBZR1* enhanced the typical xylem deficiency of *bri1-5*, implying the PgBZR1-mediated downstream event presumably resulted in different changes of gene expression, especially for xylem development. In addition, *Pgbzr1-1D* overexpression in *bri1-5* mutant background conferred stronger suppression of xylem formation than *PgBZR1* overexpression exhibited, further supporting the inhibitory role of BR during xylem differentiation in *P. ginseng*. Collectively, these results support the hypothesis that PgBZR1-mediated BR response in *P. ginseng* directs inhibition of xylem formation during secondary growth, which is different from the xylem promoting role in the model plant *Arabidopsis*.

## 3. Discussion

In the present research, we characterized components of BR signaling in *P. ginseng* and the functional role of BR signaling mediated by PgBZR1 from *P. ginseng*, the perennial medicinal plant belonging to *Araliaceae*. PgBZR1 largely mediates canonical BR signaling under the control of PgBIN2 activity through its phosphorylation status in the context of primary growth during the vegetative phase and floral development. A comprehensive study using the model plant *Arabidopsis* was conducted, as BR is known as a key positive regulator of xylem formation. However, our histological study, in combination with the pharmacological approach in *P. ginseng* and transgenic *Arabidopsis* line harboring diverse level of *PgBZR1* (*Pgbzr1-1D*) expression, revealed the opposite role of BR signaling in storage root formation of *P. ginseng*. Therefore, our study provides evidence supporting the functional diversification of BR signaling output during the secondary growth in the *Araliaceae* family.

In line with the possibility of PgBZR1 functional diversification during evolution, our phylogenetic analysis of PgBRI1, PgBIN2, and PgBZR1 also provides an interesting insight into the evolutional trajectory of the BR signaling establishment (Figure 1). During the diversification of *Magnoliophyta*, all the *BZR1 Asterids* genes were located between *Rosids* and *Poaceae*, but *Asterid* BRI1 or BIN2 were not, indicating the apparent functional divergence in BZR1-mediated BR signaling output and corresponding specificity to target genes. The mechanism involving the perception of BR by PgBRI1, consequent inactivation of PgBIN2, and nuclear accumulation of dephosphorylated PgBZR1 were highly conserved in *P. ginseng* (Figure 3, Figure 4 and Figure 5), whereas the PgBZR1-directed BR-responsive genes profile were presumably diversified in *P. ginseng* compared with other members in *Magnoliophyta* (Figure 2 and Figure 3). Further study for the identification of the direct targets of PgBZR1 and a comparative analysis of the BR-responsive genes between *P. ginseng* and *Arabidopsis* will provides important cues for the evolution of BR response in plants. In this work, we examined whether the PgBIN2-mediated phosphorylation event controls the nucleo-cytoplasmic localization of PgBZR1 (Figure 5). In a previous study, the BIN2 phosphorylation accumulated BES1 and BZR1 protein in the cytosol by 14-3-3 protein binding to phosphorylated Serine residues (14-3-3 binding domain illustrated in Figure 3B), and *bzr1-1D* mutation (P229L) completely compromised BIN2 phosphorylation [19,23]. However, despite the functionally characterized highly conserved BZR1 protein domain, including 14-3-3 binding domain, phosphorylation domain, PEST domain, and EAR motif in both of AtBZR1 and PgBZR1 (Figure 3B), the 1-1D mutation in PgBZR1 (*Pgbzr1-1D*) failed to compromise the PgBIN2 effect on subcellular protein distribution (Figure 5). These data suggest that even if Pgbzr1-1D protein is hypo-phosphorylated compared with PgBZR1, 14-3-3 binding still occurred in Pgbzr1-1D via unknown mechanisms or novel residues in Pgbzr1-1D participated in the cytosolic retention of this protein. Further study for the elucidation of the mechanism controlling PgBZR1 subcellular localization will provides insight into the fine-tuning of BR signaling transduction and/or novel regulation mechanism in other plant species. In *Arabidopsis*, BR promotes the differentiation of xylem through the action with TDIF-TDR-BIN2 interplay and finally activates BR responsive genes involved in xylem differentiation [35]. The signaling nexus from TDR-TDIF signaling directly activates BIN2 in xylem precursor cells in cambium, and then inhibits xylem formation. In the presence of BR, the inactivation of BIN2 kinase leads to an accumulation of BES1/BZR1 in the nucleus, thus facilitating xylem formation. In this study, we first demonstrated that BR signaling during the formation of the storage root of *P. ginseng* negatively affects xylem formation via PgBZR1 specific target genes (Figure 9). The BR perception and downstream signaling activation by application of BL to *P. ginseng* root were verified by the down-regulation of the well-known BR biosynthetic enzyme expressions (*PgCPD* and *PgDWF4*), indicating that the inhibitory effect of BR response on xylem formation was not a side-effect of exogenous treatment. Moreover, ectopic-expression of *PgBZR1* and *Pgbzr1-1D* in *Arabidopsis bri1-5* mutant also strongly enhanced the deficiency of xylem differentiation in both of stem and hypocotyl, pointing that PgBZR1 suppresses xylem formation. Thus, genome-wide identification and functional characterization of PgBZR1 targets during *P. ginseng* storage root development will open a new avenue in *P. ginseng* BR signaling and its application tools into enhancing secondary growth.

## 4. Materials and Methods

### 4.1. Plant Materials and Growth Conditions 

*Arabidopsis thaliana* ecotype WS2 was used as wild-type control and *bri1-5* was provided by Dr. S. Choe (Seoul National University, Seoul, Korea). *bri1-5* was used as genetic background of the transgenic lines. *Arabidopsis* seeds were germinated in media containing 1/2 Murashige and Skoog (Duchefa), 1% sucrose and 0.8% plant agar (pH 5.7) under long-day conditions (16 h light/8 h dark) at 22–24 °C. The two-year-old *P. ginseng* (Yunpoong cv.) was provided by RDA (Rural Development Administration, Jeonju, Korea) and was germinated and grown in soil (Chamgrow, Seoul, Korea) under long-day conditions (16 h light/8 h light) at 22–24 °C. 

### 4.2. Phylogeny and Heat Map Analysis 

To search for homologs of major BR signaling components (BRI1, BIN2, and BZR1) in *Magnoliophyta*, we analyzed 12 representative plant species in *Magnoliophyta* with available genome sequence on public databases including Ginsengdb (http://ginsengdb.snu.ac.kr/), Phytozome version 12.1 (https://phytozome.jgi.doe.gov/pz/portal.html), Ipomoea Genome Hub (https://ipomoea-genome.org/), Radish. Kazusa (http://radish.kazusa.or.jp/), Platycodon. theragenetex (http://platycodon.theragenetex.com/). Amino acids sequences of BRI1, BIN2, or BZR1 in *Arabidopsis* were used for blastp search via BioEdit 7.2 (https://bioedit.software.informer.com/7.2/). Sequences within top 3 hits were selected as putative BR signaling components in 12 plant species. To investigate the gene expression patterns of predicted BR signaling components in different year of *P. ginseng*, the expression levels of PgBRIs, PgGSKs, or PgBZRs were visualized using XLSTAT (https://www.xlstat.com/en/) [42,43,44].

### 4.3. Yeast-Two Hybrid Assay 

For interaction between PgGSKs and PgBZR1 in yeast cells, the AH109 was transformed with *pGADT7-PgBZR1* and *pGBKT7-PgBIN2*, *pGBKT7-PgBIL1*, or *pGBKT7-PgBIL2*. The transformed yeasts were grown on synthetic medium lacking Leu and Trp or medium lacking Leu, Trp, and His with or without 1mM 3-aminotriazole. 

### 4.4. Plasmid Construction and Transgenic Plants Analysis 

To generate transgenic plants overexpressing *PgBZR1-HA* or *Pgbzr1-1D-HA*, the full-length coding sequence (CDS) of *PgBZR1* was cloned into the pCB302ES vector that contains hemagglutinin (HA) at C-terminus [24]. The gene expression was derived by C4PPDK 35S promoter. The point mutant of *PgBZR1 (Pgbzr1-1D)* was generated using QuickChange site-directed mutagenesis kit (Stratagene). All CDSs and mutation were confirmed by DNA sequencing. These constructs were transformed into *Agrobacterium tumefaciens* strain GV3101andwere transformed into *Arabidopsis* using floral dipping method [50]. For the transient expression assay, the full length CDS of *PgBZR1* or *PgBIN2* was cloned into plant expression vectors that contain HA, GFP, or FLAG at C-terminus [24]. For phenotypic analysis, the five- to eight-week-old transgenic lines were used for analysis of stem length, silique length, and flower size. Two-week-old transgenic lines were used for dark induced hypocotyl elongation with or without 100 nM PCZ. All pictures were analyzed with imageJ for quantification.

### 4.5. Transient Expression Assay Using Arabidopsis Mesophyll Protoplast 

The 2 × 10^4^ *Arabidopsis* protoplasts were transfected with 20 μg of plasmid (*p35S:PgBZR1-HA, p35S:Pgbzr1-1D-HA, p35S:PgBZR1-GFP, p35S:Pgbzr1-1D-GFP*, or *p35S:PgBIN2-FLAG*). The transfected protoplasts were incubated for 6 h or 12 h. The proteins from protoplasts lysate that were extracted using IP buffer (50 mM Tris pH 7.5, 200 mM NaCl, 5 mM EDTA, 1% Triton X-100, 1 mM dithiothreitol, protease inhibitor cocktail (Roche, Basel, Switzerland, cat. 11697498001)) were analyzed by 10% SDS-PAGE gel and visualized with anti-HA (1:2000, Roche cat. 12013819001) or anti-FLAG (1:1000, Sigma-aldrich, St. Louis, MO, USA, cat. F3165 for primary antibody and 1:1000, Santa-cruz biotech, Dallas,TX, USA, cat. Sc-516102 for secondary antibody) using Amersham Imager 680 (cytiva). The fluorescence images were taken by epi-fluorescence microscope (Eclipse Ts2, Nikon, Tokyo, Japan). GFP or mRFP was excited using the 488 nm or 543 nm wavelength LED, respectively. The fluorescence signals between 500 nm and 520 nm were recorded for the GFP fluorescence and between 580 nm and 645 nm for mRFP fluorescence.

### 4.6. Hormone Treatment and Histological Analysis 

Two-year-old *P. ginseng* root were treated with 10 nM BL (SIGMA cat. E1641), 100 nM PCZ (SIGMA cat. 45642), or DMSO once a week for 8 weeks. Hormone treated *P. ginseng* main root samples and eight-week-old stem or hypocotyl samples of *Arabidopsis* were fixed in 3.7% formaldehyde, before dehydration with ethanol series. Samples were embedded in Surgipath paraplast (Leica, Wetzlar, Germany, cat. 39601006) at 60 °C for overnight. Sections (7 μm) were made using a HistoCore MULTICUT (Leica, Wetzlar, Germany), and counter stained with 1% Safranin-O (SIGMA, cat. S2255) and 0.5% Astra blue (Santa-cruz biochem., cat. sc-214558A) for 1 min, rinsed in distilled water, mounted in Permount mounting medium (Fisher chem., cat. SP15-100, Waltham, MA, USA) and observed using Eclipse Ts2 (Nikon). 

### 4.7. Quantitative RT-PCR

Total RNA from whole tissues of Two-week-old *P. ginseng* seedlings was extracted using easy-BLUE Total RNA Extraction kit (Intronbio, Daejeon, Korea), following the manufacturer’s instructions. Reverse transcription was carried out with 1 μg of total RNA and TOPscript ^TM^ RT DryMIX (dT18 plus) (enzynomics). qRT-PCR was performed following the instructions provided for the CFX Connect Real-Time PCR Detection System (BIO-RAD, Hercules, CALIF, USA) with the Power SYBR green PCR Master Mix (ThermoFisher, Waltham, MA, USA). PCR primers were designed using Primer express and listed at supplementary table (Tables S1 and S2) (Thermo, Waltham, MA, USA). 

## Supplementary Materials

Supplementary materials can be found at https://www.mdpi.com/1422-0067/21/24/9666/s1.

## Abbreviations

BAK1	BRI1-ASSOCIATED RECEPTOR KINASE 1
BEH	BES1/BZR1 homolog
bHLH	basic helix loop helix
BIL1	BIN2-LIKE 1
BIL2	BIN2-LIKE 2
BIN2	BRASSINOSTEROIDS INSENSITIVE 2
BKI1	BRI1 KINASE INHIBITOR 1
BL	Brassinolide
BR	Brassinosteroid
BRI1	BRASSINOSTEROIDS INSENSITIVE 1
BSU1	BRI1 SUPPRESSOR 1
BZR1/BES1	BRASSINAZOLE RESISTANCE 1/BRI1-EMS-SUPPRESSOR 1
CPD	CONSTITUTIVE PHOTOMORPHOGENESIS AND DWARFISM
DWF4	DWARF 4
ECD	Extracellular domain
ETH	Ethylene
GA	Gibberellic acid
GSK3	Glycogen synthase kinase 3
HA	Hemagglutinin
ICD	Intracellular domain
JA	Jasmonic acid
PCZ	propiconazole
PXY/TDR	PHLOEM INTERCALATED WITH XYLEM/TDIF RECEPTOR
TDIF	TRACHEARY ELEMENT DIFFERENTIATION INHIBITORY FACTOR
TF	Transcription factor
WOX4	WUSCHEL RELATED HOMEOBOX 4
WT	Wild type
Y2H	Yeast two hybrid
