# Supplementary material for: Functional Characterization of BRASSINAZOLE-RESISTANT 1 in Panax Ginseng (PgBZR1) and Brassinosteroid Response during Storage Root Formation
Source: Int J Mol Sci. 2020 Dec 18;21(24):9666. doi: 10.3390/ijms21249666 (PMC7766047; doi:10.3390/ijms21249666)
Supplement: Supplementary file 1 [file ijms-21-09666-s001.pdf]

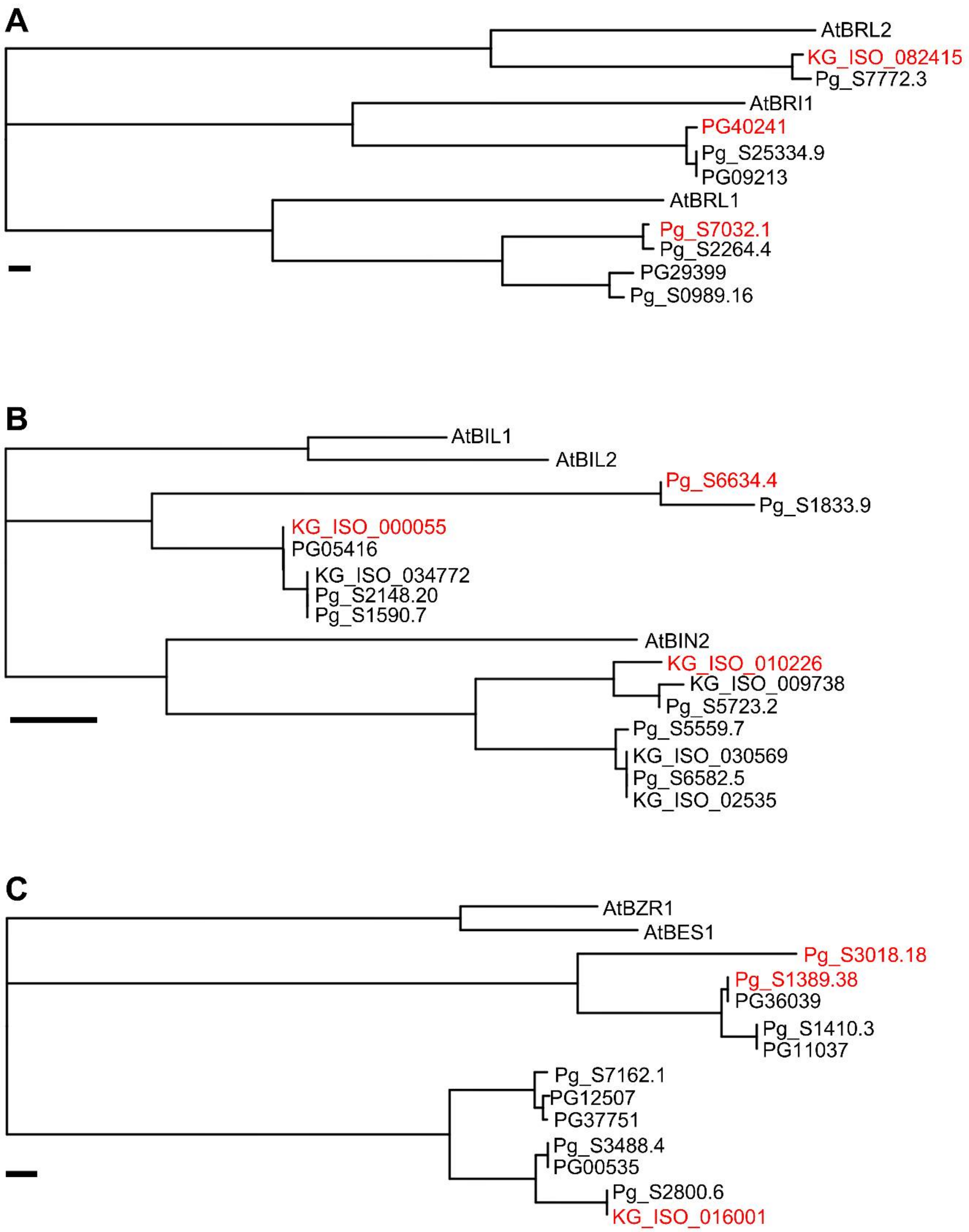


**Supplementary figure S1.** The phylogenetic analysis of predicted BR components in *P. ginseng*. A schematic diagram of the gene phylogenetic tree. Red indicates the closest components of BR signaling in *P. ginseng* comparing with *Arabidopsis*. (A) PG40241 for PgBRI1, Pg_S7032.1 for PgBRL1, and KG_ISO_082415 for PgBRL2. (B) KG_ISO_010226 for PgBIN2, Pg_S6634.4 for PgBIL1, and KG_ISO_000055 for PgBIL2. (C) KG_ISO_016001 for PgBZR1, Pg_S3018.18 for PgBZR2, and Pg_S3189.38 for PgBZR2. The phylogenetic trees were constructed with MEGA 7 program using neighbor-joining method. 3000 replicates of Bootstrap analysis. Tree scale bars, 0.01.

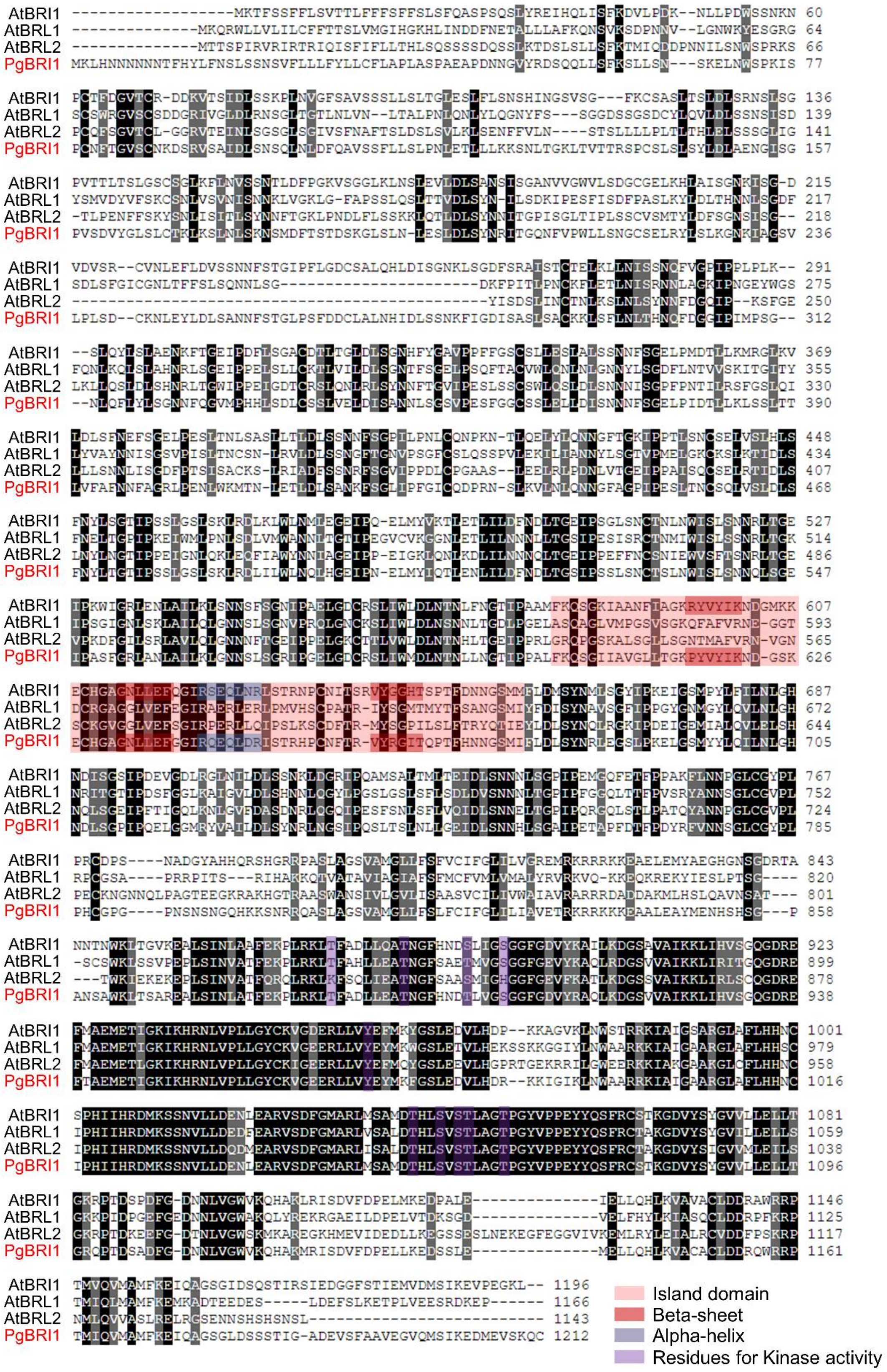


**Supplementary figure S2.** Multiple sequence alignment of PgBRI1 with *Arabidopsis* BRI1, BRL1, and BRL2. The island domain for perception of BR and the amino acid residues for kinase activity were conserved in PgBRI1. Identical amino acids were shaded in black, and similar amino acids were shaded in gray. Conserved domains and amino acid residues were indicated with different color codes.

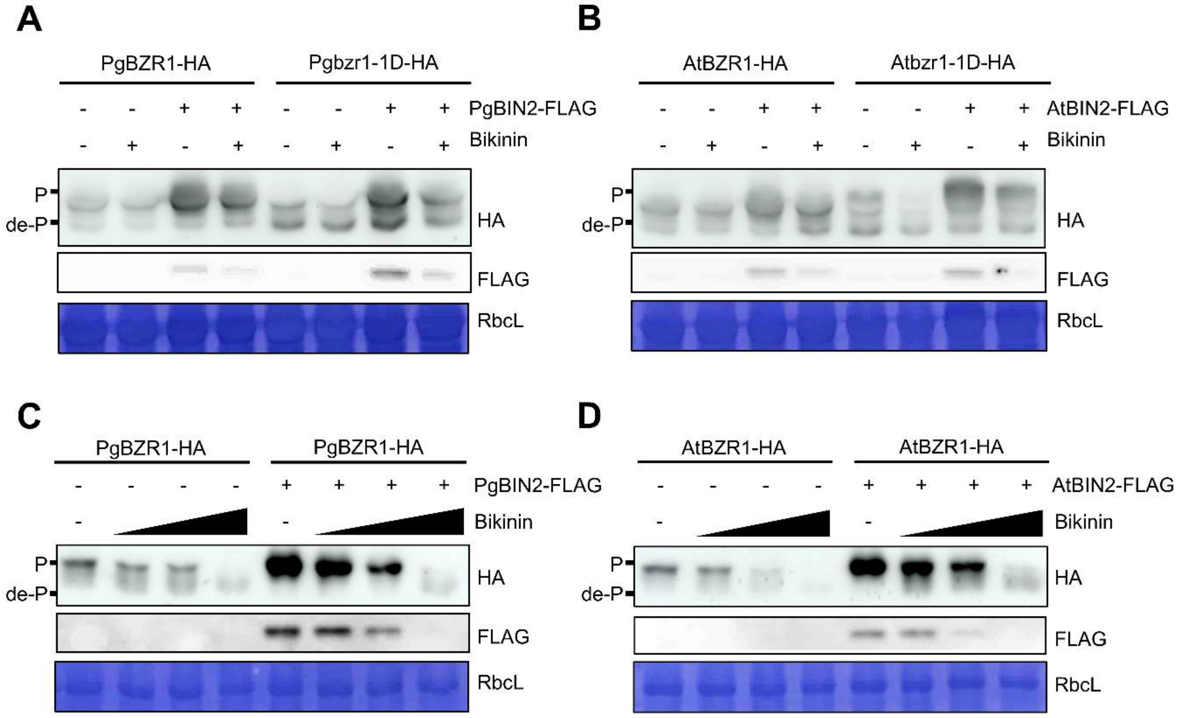


**Supplementary figure S3.** PgBIN2 shows less sensitivity to bikinin than AtBIN2. HA-tagged *BZR1* or *bzr1-1D* from *P. ginseng* or *Arabidopsis* was transfected into *Arabidopsis* protoplast with or without PgBIN2-FLAG and incubated with absence or presence of 10 μM bikinin for 6 h. The proteins were visualized with anti-HA or anti-FLAG antibodies (A, B). HA-tagged BZR1 or bzr1-1D from *P. ginseng* or *Arabidopsis* was transfected into *Arabidopsis* protoplast absence or presence of PgBIN2-FLAG or AtBIN2-FLAG, respectively. Dosage dependent effects of bikinin during 6 h incubation on BZR1 or bzr1-1D were visualized with anti-HA antibody. PgBIN2-FLAG or AtBIN2-FLAG was visualized with anti-FLAG antibody (C, D)

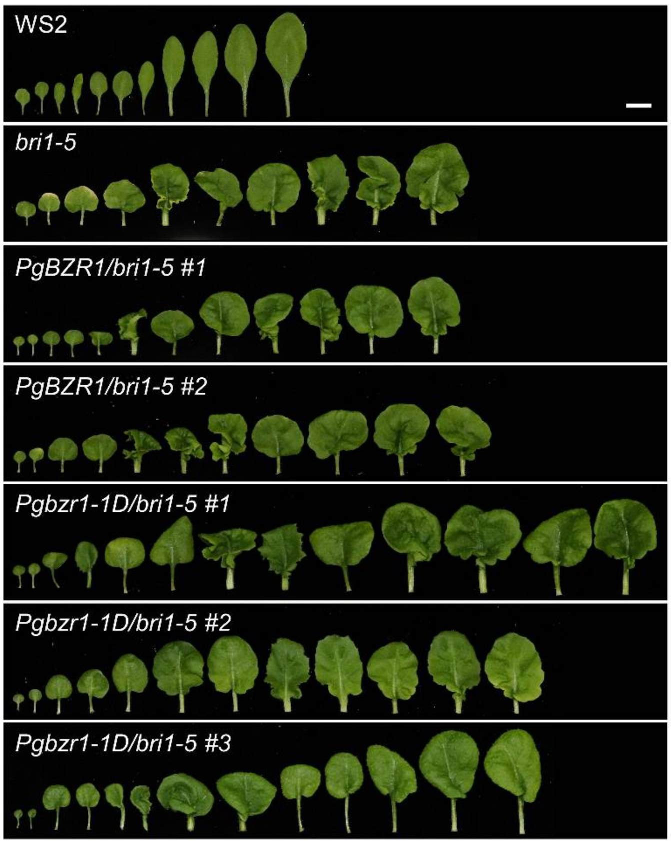


**Supplementary figure S4.** Overexpressing *Pgbzr1-1D* rescues the defective petiole phenotype of *bri1-5*. The representative rosette leaves and petiole phenotype of -week-old indicated genotypes. Scale bar, 1 cm.

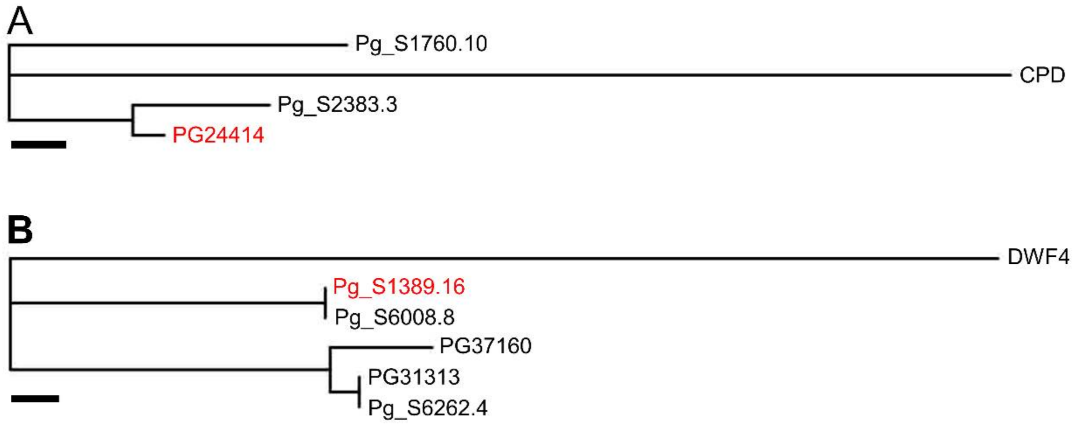


C

| Groups | Protein | Accession no. | Identification | Identity | a.a | Reference |
|---|---|---|---|---|---|---|
| **Synthesis** | PgCPD | PG24414 | Cytochrome P450 | 77% | 477 | Xu et al. 2017. |
| | PgDWF4 | Pg_S1389.16 | Cytochrome P450 | 73% | 464 | Jayakodi et al. 2018. |

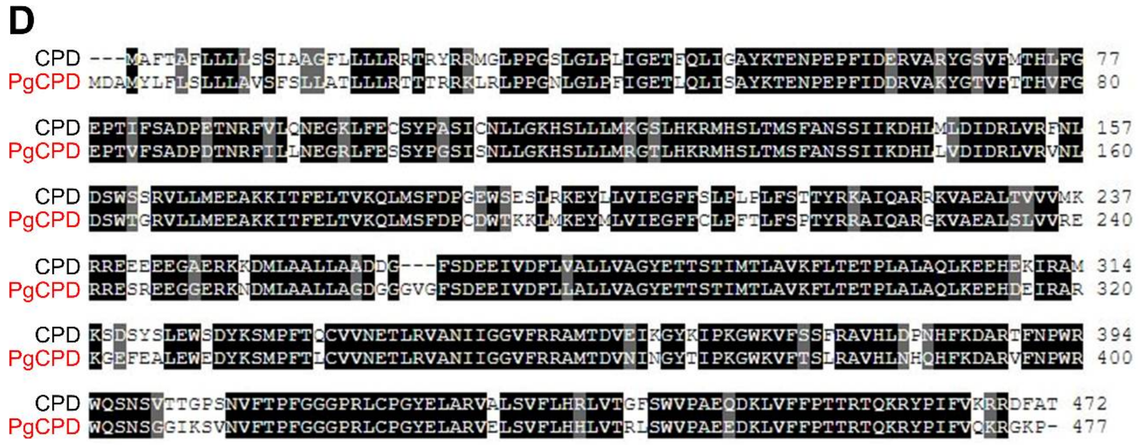


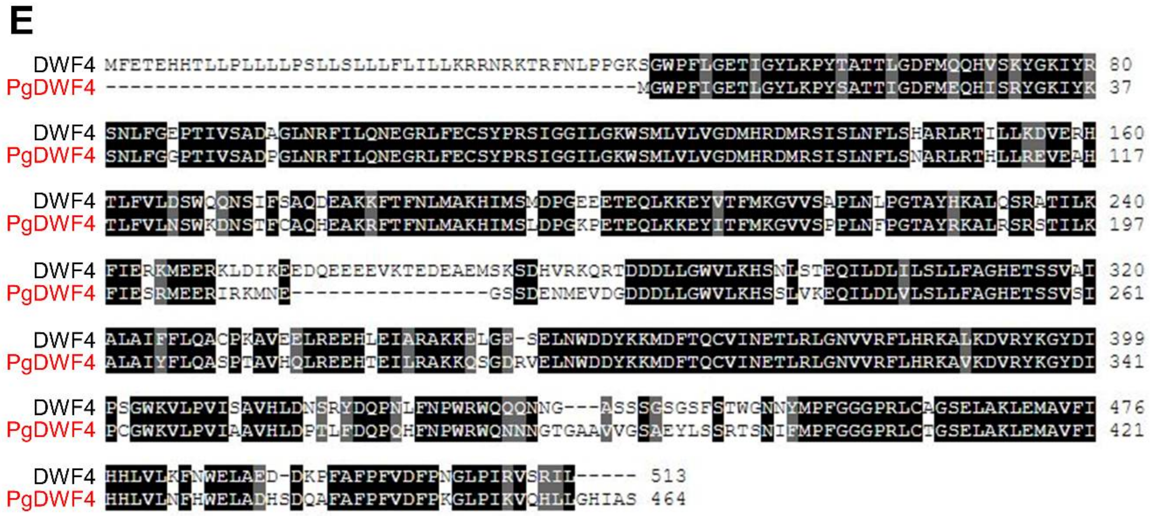


**Supplementary figure S5.** Prediction of BR synthesis related genes in *P. ginseng*. (A, B) The phylogenetic tree of *CPD* and *DWF4*. PG24414 for *PgCPD* (A) and Pg_S3189.16 for *PgDWF4* (B). (C) The accession numbers, sizes, and identities of *PgCPD* and *PgDWF4*. (D, E) Multiple sequence alignment of

PgCPD and PgDWF4 with *Arabidopsis*. ClustalX2 program was used to alignment the amino acid sequences of each components and phylogenetic trees were constructed with MEGA 7 program using neighbor-joining method. 3000 replicates of Bootstrap analysis. Tree scale bars, 0.01.

Table S1. List of primers used for qRT-PCR

| Gene | Forward | Reverse |
|---|---|---|
| qRT_PgAct | TGGCATCACTTTCTACAACG | TTTGTGTCATCTTCTCCCTGTT |
| qRT_PgCPD | AGCAATTCAGGAGGAATAAAATCG | CCGAGTCGTTGGGAAGAAAA |
| qRT_PgDWF4 | GTGGGATCAGCAGAGTACTTAAGCT | TTTGATCGGTAGGCCTTTGG |

Table S2. List of primers used for cloning

| Gene | Forward | Reverse |
|---|---|---|
| PgBZR1 | CG GGATCC ATGATGAATTGGGAAGGTGG | A AGGCCT AATCCTTGTATTCCCACTTC |
| PgBIN2 | CG GGATCC ATGGCCGACGATAAGGAGA | A AGGCCT TGTCCCTGTTGGATGCAAAA |
| PgBIL1 | GA AGATCT ATGGCCTCGTTGCCGCTG | A AGGCCT CGTCCCAGCTGGATGTGG |
| PgBIL2 | GA AGATCT ATGGTATCGTTGTCGGTGG | A AGGCCT TGTCCCGTTTGGATGGAGG |
